# Antimicrobials for the treatment of drug-resistant *Acinetobacter baumannii* pneumonia in critically ill patients: a systemic review and Bayesian network meta-analysis

**DOI:** 10.1186/s13054-017-1916-6

**Published:** 2017-12-20

**Authors:** Su Young Jung, Seung Hee Lee, Soo Young Lee, Seungwon Yang, Hayeon Noh, Eun Kyoung Chung, Jangik I. Lee

**Affiliations:** 1 Department of Pharmacy, College of Pharmacy, Seoul National University, 1 Gwanak-ro, Gwanak-gu, Seoul, 08826 Republic of Korea; 2 Research Institute of Pharmaceutical Sciences, Seoul National University, Seoul, Republic of Korea; 3 Department of Pharmacy, College of Pharmacy, Kyung Hee University, 26 Kyungheedae-ro, Dongdaemun-gu, Seoul, 02447 Republic of Korea; 4 Department of Public Health Science, Graduate School of Public Health, Seoul National University, Seoul, Republic of Korea; 5 Department of Pharmacy, College of Pharmacy, Yonsei University, Incheon, Republic of Korea

**Keywords:** Pneumonia, Critically ill patients, Antimicrobials, Drug-resistant, *Acinetobacter baumannii*, Network meta-analysis

## Abstract

**Background:**

An optimal therapy for the treatment of pneumonia caused by drug-resistant *Acinetobacter baumannii* remains unclear. This study aims to compare various antimicrobial strategies and to determine the most effective therapy for pneumonia using a network meta-analysis.

**Methods:**

Systematic search and quality assessment were performed to select eligible studies reporting one of the following outcomes: all-cause mortality, clinical cure, and microbiological eradication. The primary outcome was all-cause mortality. A network meta-analysis was conducted with a Bayesian approach. Antimicrobial treatments were ranked based on surface under the cumulative ranking curve (SUCRA) value along with estimated median outcome rate and corresponding 95% credible intervals (CrIs). Two treatments were considered significantly different if a posterior probability of superiority (*P*) was greater than 97.5%.

**Results:**

Twenty-three studies evaluating 15 antimicrobial treatments were included. Intravenous colistin monotherapy (IV COL) was selected as a common comparator, serving as a bridge for developing the network. Five treatments ranked higher than IV COL (SUCRA, 57.1%; median all-cause mortality 0.45, 95% CrI 0.41–0.48) for reducing all-cause mortality: sulbactam monotherapy (SUL, 100.0%; 0.18, 0.04–0.42), high-dose SUL (HD SUL, 85.7%; 0.31, 0.07–0.71), fosfomycin plus IV COL (FOS + IV COL, 78.6%; 0.34, 0.19–0.54), inhaled COL plus IV COL (IH COL + IV COL, 71.4%; 0.39, 0.32–0.46), and high-dose tigecycline (HD TIG, 71.4%; 0.39, 0.16–0.67). Those five treatments also ranked higher than IV COL (SUCRA, 45.5%) for improving clinical cure (72.7%, 72.7%, 63.6%, 81.8%, and 90.9%, respectively). Among the five treatments, SUL (*P* = 98.1%) and IH COL + IV COL (*P* = 99.9%) were significantly superior to IV COL for patient survival and clinical cure, respectively. In terms of microbiological eradication, FOS + IV COL (*P* = 99.8%) and SUL (*P* = 98.9%) were significantly superior to IV COL.

**Conclusions:**

This Bayesian network meta-analysis demonstrated the comparative effectiveness of fifteen antimicrobial treatments for drug-resistant *A. baumannii* pneumonia in critically ill patients. For survival benefit, SUL appears to be the best treatment followed by HD SUL, FOS + IV COL, IH COL + IV COL, HD TIG, and IV COL therapy, in numerical order.

**Electronic supplementary material:**

The online version of this article (doi:10.1186/s13054-017-1916-6) contains supplementary material, which is available to authorized users.

## Background

Nosocomial pneumonia is a leading cause of death in critically ill patients [1, 2]. The mortality rates associated with hospital-acquired pneumonia (HAP) or ventilator-associated pneumonia (VAP) in intensive care units (ICU) range from 38% to 70% or higher [3, 4]. One of the most common pathogens of nosocomial pneumonia is *Acinetobacter baumannii* (*A. baumannii*). Because *A. baumannii* exhibits relatively high virulence and antimicrobial resistance compared with other organisms, the prevalence of multidrug-resistant (MDR) or extensively drug-resistant (XDR) *A. baumannii* has kept increasing to at least 80% in past decades [5]. One of the important risk factors for MDR/XDR-bacterial infections in ICU is an inappropriate antibiotic therapy [6]. Considering the paucity of clinical data on the comparative effectiveness of various antimicrobial treatments for MDR/XDR *A. baumannii* pneumonia, it is pressing to accumulate clinically reliable scientific evidence to guide the selection of an optimal antimicrobial treatment for the infection.

Colistin-based, sulbactam-based and tigecycline-based antimicrobial treatments are currently considered the reserved treatment options for MDR/XDR *A. baumannii* infections [7, 8]. However, the clinical data comparing the antimicrobial treatments adequately with respect to the effectiveness and safety of the treatment of such infections, particularly nosocomial pneumonia, are inconsistent owing to small sample sizes, and substantial between-study heterogeneity [9–13]. In addition, to date there is no consensus based on strong evidence to confirm the therapeutic superiority of a monotherapy or combination therapy and clinical preferences among various antimicrobial combination regimens [9, 10]. Therefore, clinicians are still facing challenges to apply evidence-based pharmacotherapy in clinical practice for the treatment of nosocomial pneumonia.

Currently available studies have compared the effectiveness of different antimicrobial treatment options only in pairs within the studies [14–16]. Although the results from those studies are informative, the relative effectiveness throughout a variety of therapeutic options remains unknown. Network meta-analysis (NMA) is a relatively novel meta-analysis strategy that integrates both direct evidence from pairwise comparisons within a study and indirect evidence from common-comparator comparisons across the studies [17, 18]. Compared with conventional meta-analyses, NMA allows comparisons across multiple treatments simultaneously even when the treatments were not directly compared in previous studies. Furthermore, NMA using a Bayesian approach uniquely provides the probability estimates that enable clinicians to make an intuitive pharmacotherapy decision [19]. The Bayesian approach allows us to adopt the strengths from data across multiple studies and does not require an assumption of normal distribution [20]. Therefore, the approach is more favorable than the frequentist approach when small numbers of studies are included in each pair of comparisons.

The aim of this study was to evaluate the comparative effectiveness of currently available antimicrobial options, including monotherapy and combination therapy, for the treatment of critically ill patients with nosocomial pneumonia caused by MDR/XDR *A. baumannii*, using a Bayesian NMA approach.

## Methods

A systematic review and the Bayesian NMA were performed in accordance with the Preferred Reporting Items for Systematic Reviews and Meta-analyses (PRISMA) extension statement for network meta-analyses [21].

### Search strategy

A comprehensive literature search was performed using the electronic databases of PubMed, Embase, and the Cochrane Central Register of Controlled Trials from the inception of each database to 31 March 2017. The literature search was limited to human studies without language restrictions. In order to identify appropriate articles that evaluated the antimicrobial treatments for patients with nosocomial pneumonia caused by MDR/XDR pathogens, the following text words and medical subject heading (MeSH) terms were used: “pneumonia”, “HAP”, or “VAP”; “drug resistant”, “carbapenem-resistant”, “MDR”, or “XDR”; “aminoglycoside”, “carbapenems”, “colistin”, “fosfomycin”, “glycopeptide”, “glycylcycline”, “polymyxin”, “rifampin”, “sulbactam”, or “tigecycline”. In addition, proceedings from relevant conferences and the references that were listed in all retrieved articles were also manually searched to ensure a complete identification of all eligible studies.

### Selection criteria

The types of study included in the analysis were randomized controlled trials (RCTs) and observational studies in which antimicrobial agents were compared in the treatment of critically ill adult patients with HAP or VAP caused by MDR/XDR *A. baumannii*. Studies were included if they evaluated at least one of the following three outcomes with clear definitions: all-cause mortality, clinical cure, or microbiological eradication. All-cause mortality, defined as the incidence of deaths from any cause in ICU within the follow-up duration of approximately 30 days, was chosen as the primary outcome variable for the comparison of antimicrobial effectiveness. If all-cause mortality in the ICU was not available in the studies, in-hospital mortality data were used. Clinical cure and microbiological eradication were selected as the secondary outcome variables. Clinical cure was defined as a resolution of signs and symptoms of pneumonia by the end of therapy, whereas microbiological eradication was defined as a confirmed negative result in a follow-up bacterial culture by the end of therapy.

Excluded studies are as follows: (1) case series, (2) studies that enrolled pediatric patients, (3) studies that enrolled patients with community-acquired pneumonia, and (4) studies in which less than half of study population had pneumonia.

### Quality assessment and data extraction

Two investigators (SYJ and SHL) independently screened the titles and abstracts of the articles, and reviewed their full texts based on the pre-specified selection criteria. Any inconsistencies between the two investigators were resolved by extensive discussion with the third investigator (HN). Quality assessment of included studies was performed by two investigators (SYJ and SHL), and was crosschecked by the third investigator (SY). The quality of included RCTs was assessed using the Cochrane Collaboration Risk of Bias Tool [22]. The quality of observational studies was evaluated using the Newcastle-Ottawa Scale (NOS) [23]. Studies with an NOS score <7 were considered at high risk of bias [24, 25]. After excluding the studies with high risk of bias, the remaining studies were included in the final analyses. Two investigators (SYJ and SHL) independently extracted the following data from each study using a standardized extraction form: year of publication, type of study design (i.e., RCT or observational study), patient population characteristics, specific antimicrobial therapy with dosing strategy as either an intervention or a comparator, and outcome measurements with their definitions. Antimicrobials administrated at greater than standard dosage for the treatment of MDR/XDR *A. baumannii* pneumonia were separately categorized as high-dose regimens for each antimicrobial agent, as referred to in previous studies [26–28].

### Data synthesis and analysis

Pairwise meta-analyses were initially performed for direct comparisons between different antimicrobial treatments using Stata software (version 13.0, StataCorp, College Station, TX, USA). Homogeneity and consistency were assumed in drawing valid conclusions from NMA analyses [29]. Homogeneity assumption was satisfied when the magnitude of heterogeneity within direct pairwise comparisons was acceptable. Heterogeneity of the treatment effects across trials in each pair was examined by the *Q* test and quantified using the *I* ^2^ statistic. The *I* ^2^ values < 40%, 40%–75%, and > 75% accompanied by a *p* value <0.10 from the *Q* test were considered mild, moderate, and high heterogeneity, respectively [30]. A consistency assumption referring to the lack of disagreements between direct and indirect comparisons was required to be met in integrating direct and indirect evidence in the NMA [31]. Inconsistency in the entire network of each outcome was assessed by either the Lu-Ades model or design-by-treatment interaction model, according to a configuration of the loop in the network [32–34]. The consistency assumption was rejected when the *p* value of the inconsistency test was < 0.05. Publication bias was also evaluated with a funnel plot and Egger’s test if ten or more studies were included in each pairwise meta-analysis [30].

The Bayesian NMA was performed for multiple comparisons using WinBUGS (version 1.4.3, MRC Biostatistics Unit, Cambridge, UK). Both fixed-effect and random-effect models were fitted. The best model was chosen based on the deviance information criterion (DIC) that suggests a significantly better fit of the model with a value lowered by 2–3 points [35]. Markov chain Monte Carlo (MCMC) samplers were run in WinBUGS using three chains with different initial values. Non-informative priors were used to produce posterior distribution for the treatment effects, allowing the data to dominate the final estimates (vague normal distribution with mean of 0 and variance of 0.0001). There were 10,000 updates generated for each set of the chains, and the first 10,000 iterations were discarded as the burn-in phase. Brooks-Gelman-Rubin diagnostic plots were used to verify the convergence of the MCMC simulations [36].

The estimates of Bayesian NMA were reported as rank probabilities to identify the relative rankings of antimicrobial treatments based on the surface under the cumulative ranking curve (SUCRA), ranging from 0% (statistically certain to be the worst treatment) to 100% (statistically certain to be the best treatment) [37]. Because SUCRA rankings could exaggerate the small differences since those are relative values, the estimates of median outcome rates were also reported with the corresponding 95% credible intervals (CrIs) to specify the absolute magnitude of therapeutic effectiveness. Then, Bayesian posterior probabilities of superiority were calculated to identify a significant difference between an individual treatment and a common comparator. The probabilities of superiority (*P*) indicated the probabilities of the odds ratio (OR) being < 1 for all-cause mortality and > 1 for clinical cure and microbiological eradication. The treatment with *P* > 97.5% or *P* < 2.5% was considered statistically superior or inferior to the comparator, respectively [38–40]. Although whether or not a confidence interval (CI) for the OR not crosses 1 determines a statistically significant difference in frequentist statistics, the concept of posterior probability in Bayesian statistics was used to demonstrate the certainty of comparative results [41–43].

Sensitivity analyses were performed to evaluate the robustness of the Bayesian NMA results. Although most patients included in this analysis had drug-resistant *A. baumannii* pneumonia, the comparative effectiveness of antimicrobial treatments could be affected by type(s) of infection other than pneumonia or causative pathogen(s) other than *A. baumannii*. Therefore, two sensitivity analyses were performed exclusively using the studies in which all patients had pneumonia or were infected by *A. baumannii*, respectively.

## Results

### Study selection and quality assessment

**Fig. 1:**
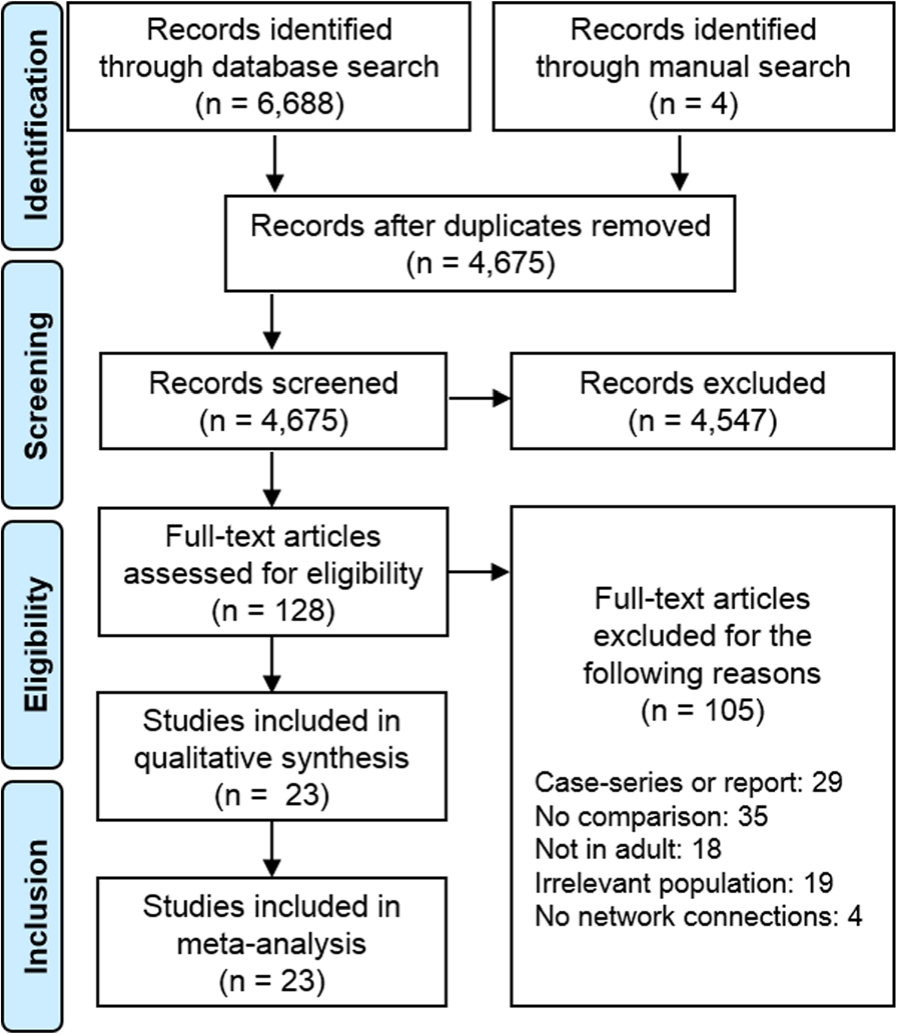
Study selection process according to the Preferred Reporting Items for Systematic Reviews and Meta-analyses guideline

A total of 6688 articles were identified through the electronic database search. Additional 4 records were identified through a manual search by reviewing conference proceedings and the reference lists of retrieved articles. After removing 2017 duplicates, 4675 articles were assessed for relevance by screening the title and abstract. Afterward, 128 relevant articles were screened for eligibility by full-text evaluations. Finally, 23 articles that met the inclusion criteria were included in our Bayesian NMA (Fig. 1). Those 23 articles consist of four RCTs [44–47], three prospective observational studies [48–50], and sixteen retrospective studies [51–66]. A PRISMA extension checklist for reporting systematic reviews comparing multiple treatments involving NMA is shown in Additional file 1: Table S1.

The results of quality assessment for the included studies are shown in Additional file 2: Table S2 and Table S3. None of the RCTs had high risk of bias for sequence generation, addressing outcome data, selective reporting, or funding source. However, two studies had high risk of bias for double blinding. Bias assessment for allocation concealment was difficult owing to insufficient information available in most studies. All observational studies had a total bias score of at least 7 points based on the NOS, which indicates a low risk of bias.

### Study characteristics

**Fig. 2:**
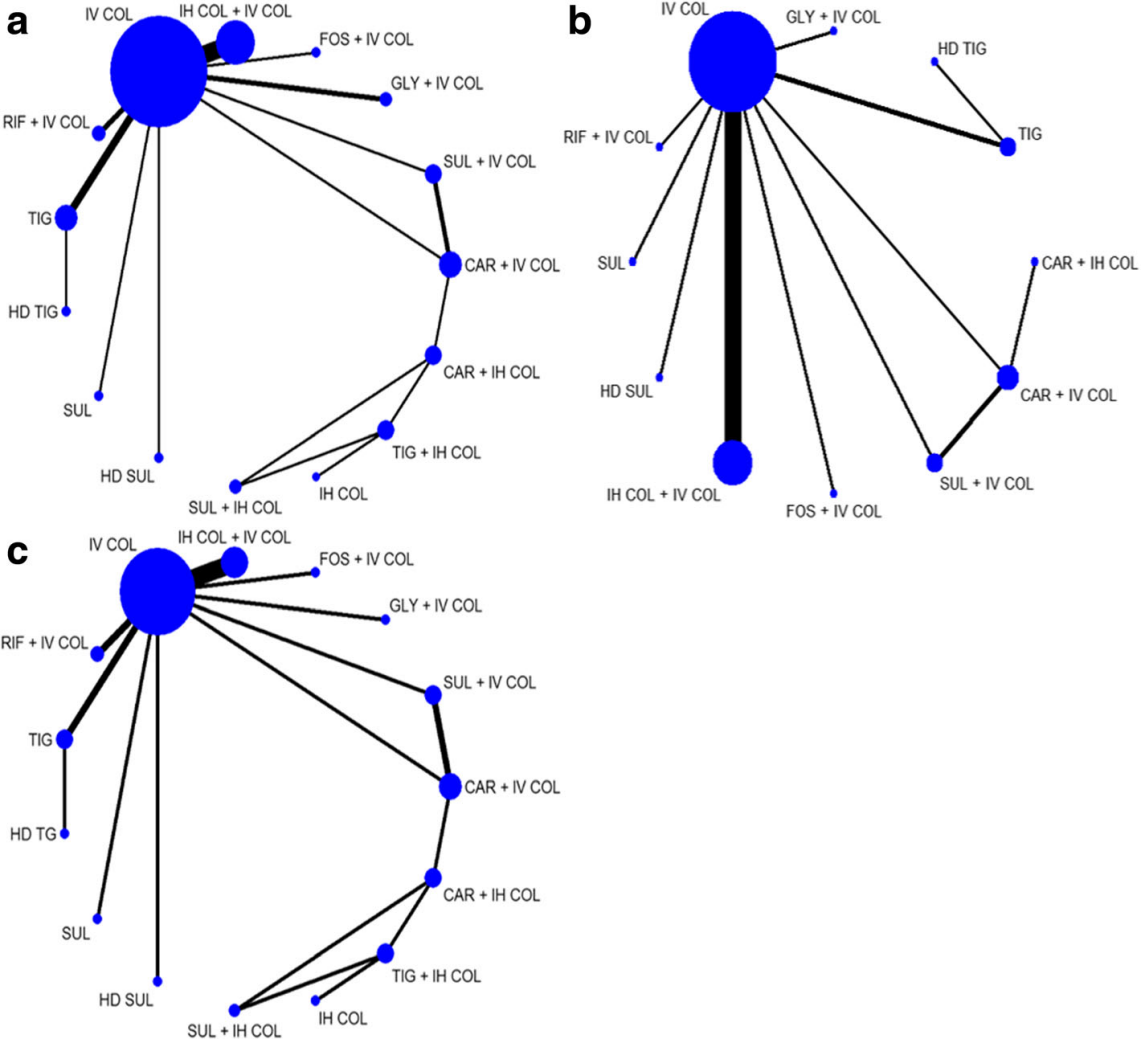
Networks of direct comparisons. **a** All-cause mortality. **b** Clinical cure. **c** Microbiological eradication. The size of the nodes and the thickness of the lines indicate the sample size and number of trials, respectively. Lines do not connect nodes when there were no head-to-head trials between two treatments. *Abbreviations*: *CAR* carbapenem (imipenem or meropenem), *COL* colistin, *FOS* fosfomycin, *GLY* glycopeptide (vancomycin or teicoplanin), *HD* high-dose, *IH* inhaled, *IV* intravenous, *RIF* rifampin, *SUL* sulbactam, *TIG* tigecycline

The Bayesian NMA was performed using 23 studies that consist of a total of 2118 adult patients. Table 1 shows the characteristics of the studies including patient population, antimicrobial therapy with a total daily dose, and outcomes of each study. The NMA evaluated 15 different antimicrobial treatments, including intravenous colistin (IV COL), sulbactam (SUL), and tigecycline (TIG) as monotherapy or combination therapy. Among them, SUL and TIG administrated at their higher doses were separately categorized as high-dose (HD) regimens: HD SUL defined as a total daily dose of 9 g/day or higher and HD TIG as a total daily dose of 200 mg/day after a loading dose of 200 mg [49, 52]. Across the studies analyzed, the mean (or median) age of the patients ranged from 48 to 82 years, and the mean Acute Physiology and Chronic Health Evaluation II (APACHE II) score from 12 to 24. Most studies reported two-arm comparisons except for two studies with three-arm comparisons. Figure 2 presents the network plots of direct comparisons for each outcome, showing predominant pairwise comparisons of antimicrobials with IV COL. IV COL also served as a bridge node to construct the network with a closed loop, allowing indirect comparisons within the network. Hence, IV COL was selected as a common comparator in the Bayesian NMA.

**Table 1:** Characteristics of the studies included in the Bayesian network meta-analysis

1st Author, publication year	Infection type (%)	Pathogen (%)	Antimicrobial therapy^a^ (total daily dose)	Mean age (years)	Disease severity score^b^	Outcomes (*n*/*N*)		
						All-cause mortality	Clinical cure	Microbiological eradication
Abdellatif, 2016 [44]	VAP 100	MDR; AB 55, PA 21, etc. 24	CAR (3 g), IV COL (9 MIU)	53	SOFA: 6.5	18/76	55/76	57/76
			CAR (3 g), IH COL (12 MIU)	50	SOFA: 7.0	20/73	49/73	55/73
Amin, 2013 [48]	VAP (or HAP) 100	MDR; AB 65, PA 25, KP 10	IV COL (3–8 MIU)	61	19.1	5/12	7/12	NR
			IH COL (3–-8 MIU), IV COL (4 MIU)	56	18.1	8/28	22/28	
Aydemir, 2013 [45]	VAP 100	CR; AB 100	IV COL (9 MIU)	63	18.0	16/22	9/22	13/22
			RIF (600 mg), IV COL (9 MIU)	58	20.1	13/21	11/21	15/21
Betrosian, 2008 [49]	VAP 100	MDR; AB 100	IV COL (9 MIU)	67	14.0	5/15	9/15	10/15
			HD SUL (9 g)	72	14.0	3/13	9/13	8/13
Chuang, 2014 [51]	VAP (or HAP) 100	MDR; AB 100	IV COL (2.5–5.0 mg/kg)	64	21.6	37/84	NR	NR
			TIG (100 mg)	64	22.0	51/84		
De Pascale, 2014 [52]	VAP 100	MDR, XDR; AB 44, KP 48, etc. 32	TIG (100 mg)	65	SOFA: 7.8	20/30	10/30	7/30
			HD TIG (200 mg)	61	SOFA: 7.4	16/33	19/33	12/33
Demirdal, 2016 [53]	VAP (or HAP) 100	MDR; AB 100	IV COL (300 mg)	63	NR	38/80	30/80	40/80
			IH COL (300 mg), IV COL (150 mg)	67		23/43	16/43	20/43
Doshi, 2013 [54]	VAP (or HAP) 100	MDR; AB 64, PA 56, KP 12	IV COL (5 mg/kg)	57	24.0	19/27	20/51	11/27
			IH COL (150 or 300 mg), IV COL (5 mg/kg)	61	22.4	6/15	24/44	8/18
Durante Mangoni, 2013 [46]	VAP (or HAP) 78^c^	XDR; AB 100	IV COL (6 MIU)	61	SAPSII: 39.0	45/105	NR	47/105
			RIF (600 mg), IV COL (6 MIU)	62	SAPSII: 40.8	45/104		63/104
Frantzeskaki, 2013 [50]	VAP 100	MDR; AB 100	CAR (6 g), IV COL (9 MIU)	68	12.0	5/8	6/8	6/8
			SUL (8 g), IV COL (9 MIU)	63	13.0	8/16	13/16	13/16
Garnacho-Montero, 2013 [55]	VAP 86^d^	CR; AB 100	IV COL (6 or 9 MIU)	63	19.0	14/28	20/28	15/23
			GLY (TDM), IV COL (6 or 9 MIU)	54	16.0	14/29	17/29	13/24
Hsieh, 2014 [56]	VAP 100	XDR; AB 100	IH COL (4 MIU CMS)	82	16.4	3/9	NR	7/9
			IH COL (4 MIU CMS), TIG (100 mg)	79	17.8	10/29		23/29
Kalin, 2012 [57]	VAP 100	MDR; AB 100	IV COL (5 or 10 mg/kg)	48	22.0^f^	7/16	6/16	11/16
			IH COL (150 mg), IV COL (5 or 10 mg/kg)	51	22.0^e^	16/29	4/29	22/29
Khawcharoenporn, 2014 [58]	VAP 55, HAP 45	XDR; AB 100	CAR (3 g), IH COL (160 mg CMS)	75^e^	18.0^e^	18/30	NR	16/22
			TIG (100 mg), IH COL (160 mg CMS)	75^e^	20.0^e^	23/43		25/38
			SUL (6 g), IH COL (160 mg CMS)	75^e^	18.0^e^	60/93		59/70
Kim, 2016 [59]	VAP 73, HAP 27	MDR or XDR; AB 100	IV COL (300 mg)	67^e^	SOFA: 10.0^e^	16/40	19/40	12/40
			TIG (100 mg)	72^e^	SOFA: 9.5^e^	14/30	14/30	7/30
Kofteridis, 2010 [60]	VAP100	MDR; AB 77, PA 9, KP 14	IV COL (9 MIU)	62	17.7	18/43	14/43	17/43
			IH COL (2 MIU), IV COL (9 MIU)	62	17.0	10/43	23/43	19/43
Korbila, 2010 [61]	VAP 100	MDR; AB 76, PA 18, KP 6	IV COL (6 MIU)^f^	61	19.2	18/38	22/38	NR
			IH COL (2 MIU)^f^, IV COL (7 MIU)^f^	59	17.4	24/60	46/60	
Kwon, 2014 [62]	HAP 75^g^	XDR; AB 100	IV COL (75–300 mg CMS)	59	NR	17/39	19/39	18/39
			TIG (50–100 mg)	60		9/16	7/16	2/16
Petrosillo, 2014 [63]	VAP 64^h^	MDR; AB 57, PA 18, KP 17	IV COL (6 MIU)	65^f^	20.0^e^	17/61	NR	NR
			GLY (VAN 2 g; TEI 400 mg), IV COL (6 MIU)	68^f^	21.0^e^	14/42		
Sirijatuphat, 2014 [47]	VAP (or HAP) 78^i^	CR; AB 100	IV COL (5 mg/kg)	69	21.9	27/47	26/47	38/47
			FOS (8 g), IV COL (5 mg/kg)	67	23.0	22/47	28/47	46/47
Tumbarello, 2013 [64]	VAP 100	XDR; AB 62, PA 25, KP 13	IV COL (0.1 MIU/kg)	66^e^	SOFA: 8.0^e^	48/104	57/104	42/84
			IH COL (3 MIU), IV COL (0.1 MIU/kg)	64^e^	SOFA: 7.0^e^	45/104	72/104	52/82
Yilmaz, 2015 [65]	VAP 100	MDR or XDR; AB 100	IV COL (225 or 300 mg CMS)	60	SAPSII: 43.8	7/17	13/17	9/17
			CAR (IMI 2 g; MERO 3 g), IV COL (225 or 300 mg CMS)	60	SAPSII: 50.7	16/33	21/33	21/33
			SUL (3 g), IV COL (225 or 300 mg CMS)	71	SAPSII: 51.0	14/20	11/20	12/20
Zalts, 2016 [66]	VAP 100	CR; AB 100	IV COL (6 MIU)	57	17.8	17/66	31/66	17/33
			SUL (4 g)	50	17.2	3/32	18/32	14/17

*Abbreviations*: *AB Acinetobacter baumannii*, *CAR* carbapenem, *COL* colistin (mostly in colistin base activity (CBA) units), *CR* carbapenem-resistant, *FOS* fosfomycin, *GLY* glycopeptide, *HAP* hospital-acquired pneumonia, *HD* high dose, *IH* inhaled, *IV* intravenous, *IMI* imipenem, *KP Klebsiella pneumoniae*, *MDR* multidrug-resistant, *MERO* meropenem, *MIU* million international units, *n/N* number of outcome events/number of patients in each intervention, *NR* not reported, *PA Pseudomonas aeruginosa*, *RIF* rifampin, *SAPS* Simplified Acute Physiology Score, *SOFA* Sepsis-Related Organ Failure Assessment score, *SUL* sulbactam (frequently used with ampicillin but described only as SUL in the table), *TDM* therapeutic drug monitoring, *TEI* teicoplanin, *TIG* tigecycline, *VAN* vancomycin, *VAP* ventilator-associated pneumonia, *XDR* extensively drug-resistant

^a^COL 1 MIU ≒ 30 mg of colistin base activity (CBA) ≒ 80 mg of colistimethate (CMS)

^b^Values are mean Acute Physiology and Chronic Health Evaluation II score unless otherwise indicated

^c^Other types: bloodstream infection 20%, complicated intra-abdominal infection 2%

^d^Other types: bloodstream infection 14%

^e^Median value

^f^Mean value

^g^Other types: bloodstream infection 13%, wound infection 5%, peritonitis 3%, urinary tract infection 2%, biliary tract infection 2%

^h^Other types: bloodstream infection 19%, etc. 17%

^i^Other types: bloodstream infection 5%, urinary tract infection 5%, soft skin tissue infection 3%, intra-abdominal infection 6%, etc. 3%

### Assessment of heterogeneity, inconsistency, and model fit

Table 2 presents the results of heterogeneity in direct pairwise comparisons involving at least two studies. Overall, no statistically significant heterogeneity was observed in most direct pairwise comparisons for each outcome (*I* ^2^ < 40%, *p* > 0.01) except for two comparison pairs. Comparison pairs of SUL plus IV COL (SUL + IV COL) versus carbapenem plus IV COL (CAR + IV COR) for all-cause mortality (*I* ^2^ = 43.2%), and IV COL versus TIG for microbiological eradication (*I* ^2^ = 53.4%) showed moderate heterogeneity, but was not statistically significant (*p* > 0.10). Publication bias was not evaluated because the number of studies included in each pair was fewer than 10. There was no evidence of significant inconsistency in the entire network of each outcome (*p* > 0.05; Additional file 3: Table S4). Inconsistency was assessed based on the design-by-treatment interaction model because a triangle-loop of each network was formed by one three-arm and one two-arm study in common. All Bayesian NMA estimates were derived from fixed-effect models because of their better fit than that of random-effect models for all three outcomes (all-cause mortality, DIC_fixed_ = 84 versus DIC_random_ = 86; clinical cure, 68 versus 70; microbiological eradication, 71 versus 73).

**Table 2:** Direct pairwise comparisons and heterogeneity

Comparison	Pairwise OR (95% CI)	Number of events	Number of patients	Number of studies	Heterogeneity test	
					*I* ^2^ (%)^a^	*p* value^b^
All-cause mortality						
IV COL vs.						
GLY + IV COL	1.13 (0.59–2.19)	59	160	2	0.0	0.63
IH COL + IV COL	0.80 (0.58–1.11)	285	642	7	18.2	0.29
RIF + IV COL	0.94 (0.57–1.56)	119	252	2	0.0	0.47
TIG	1.73 (1.08–2.78)	144	293	3	0.0	0.78
CAR + IV COL vs.						
SUL + IV COL	1.59 (0.60–4.20)	43	77	2	43.2	0.18
Clinical cure						
IV COL vs.						
CAR + IV COL	0.81 (0.37–1.76)	199	248	2	0.0	0.46
IH COL + IV COL	1.60 (1.05–2.46)	363	695	7	38.0	0.14
TIG	0.91 (0.43–1.89)	59	125	2	0.0	0.83
CAR + IV COL vs.						
SUL + IV COL	0.60 (0.17–2.11)	51	77	2	11.3	0.29
Microbiological eradication						
IV COL *vs.*						
IH COL + IV COL	1.28 (0.88–1.57)	242	465	5	0.0	0.73
RIF + IV COL	1.87 (1.13–3.10)	138	252	2	0.0	0.90
TIG	0.45 (0.18–1.11)	39	125	2	53.4	0.14
CAR + IV COL vs.						
SUL + IV COL	1.36 (0.45–4.10)	52	77	2	0.0	0.95

*Abbreviations*: *CAR* carbapenem (imipenem or meropenem), *CI* confidence interval, *COL* colistin, *FOS* fosfomycin, *GLY* glycopeptide (vancomycin or teicoplanin), *HD* high dose, *IH* inhaled, *IV* intravenous, *OR* odds ratio, *RIF* rifampin, *SUL* sulbactam, *TIG* tigecycline, *vs.* versus

^a^Quantified value of OR variation attributable to heterogeneity

^b^ *p* value from *Q* test based on chi-square statistic

### Bayesian NMA ranking

**Fig. 3:**
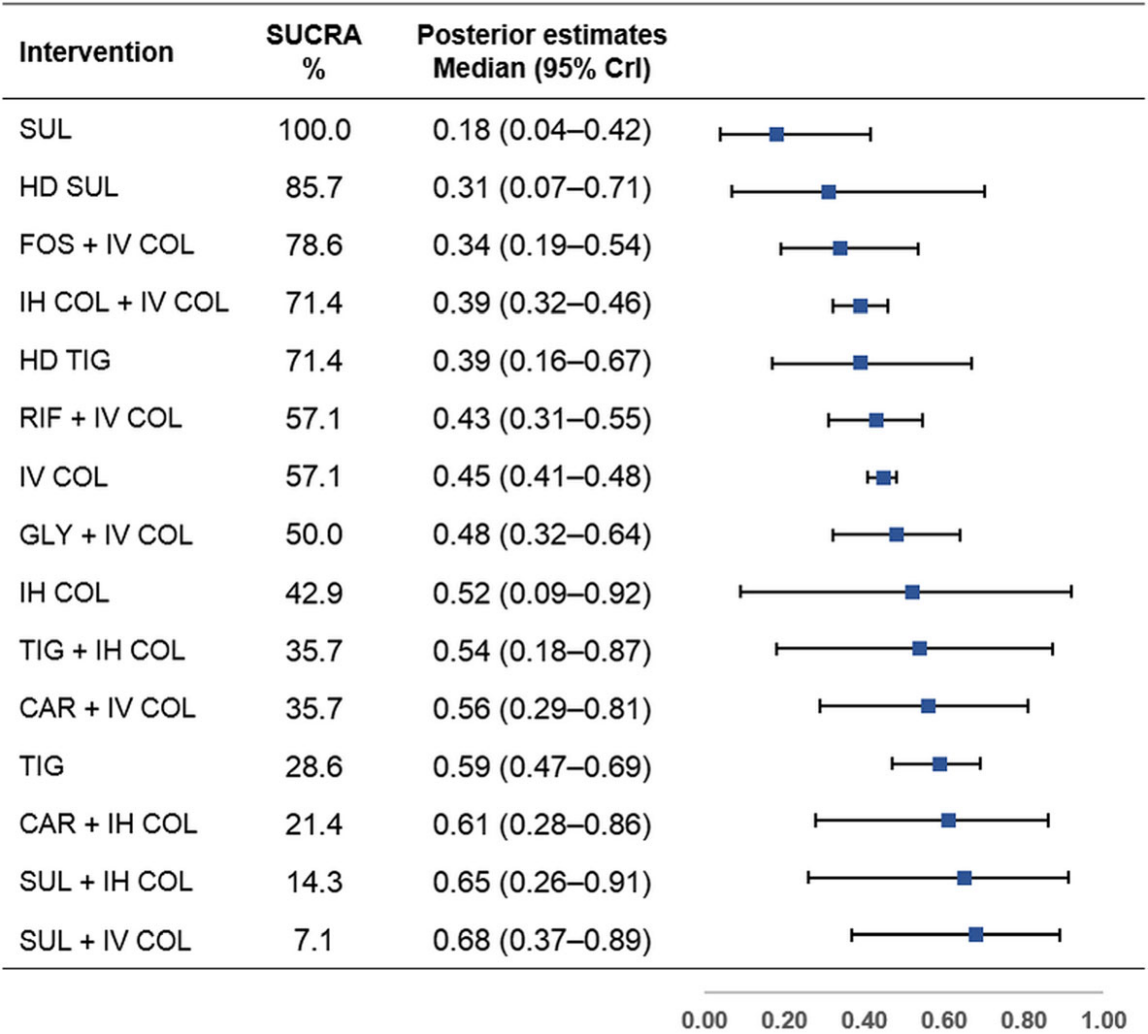
Surface under the cumulative ranking curve (SUCRA) rankings and posterior estimates of treatment effect on all-cause mortality. Greater SUCRA value indicate higher probability of being the best treatment for reducing all-cause mortality. *Abbreviations*: *CAR* carbapenem (imipenem or meropenem), *COL* colistin, *CrI* credible interval, *FOS* fosfomycin, *GLY* glycopeptide (vancomycin or teicoplanin), *HD* high-dose, *IH* inhaled, *IV* intravenous, *RIF* rifampin, *SUL* sulbactam, *TIG* tigecycline

The SUCRA rank probabilities and Bayesian posterior estimates of the effect of various antimicrobial treatments on all-cause mortality are presented in Fig. 3. SUL monotherapy ranked first for reducing all-cause mortality (SUCRA, 100.0%; median outcome rate 0.18, 95% CrI 0.04–0.42) among the 15 antimicrobial treatments. Four treatments, in addition to SUL, ranked higher than IV COL (57.1%; 0.45, 0.41–0.48) and included HD SUL (85.7%; 0.31, 0.07–0.71), fosfomycin plus IV COL (FOS + IV COL, 78.6%; 0.34, 0.19–0.54), inhaled COL plus IV COL (IH COL + IV COL, 71.4%; 0.39, 0.32–0.46), and HD TIG (71.4%; 0.39, 0.16–0.67). Rifampin plus IV COL (RIF + IV COL, 57.1%; 0.43, 0.31–0.55) ranked the same as IV COL. SUL + IV COL (7.1%; 0.68, 0.37–0.89) ranked the lowest among the 15 different antimicrobial treatments.

**Fig. 4:**
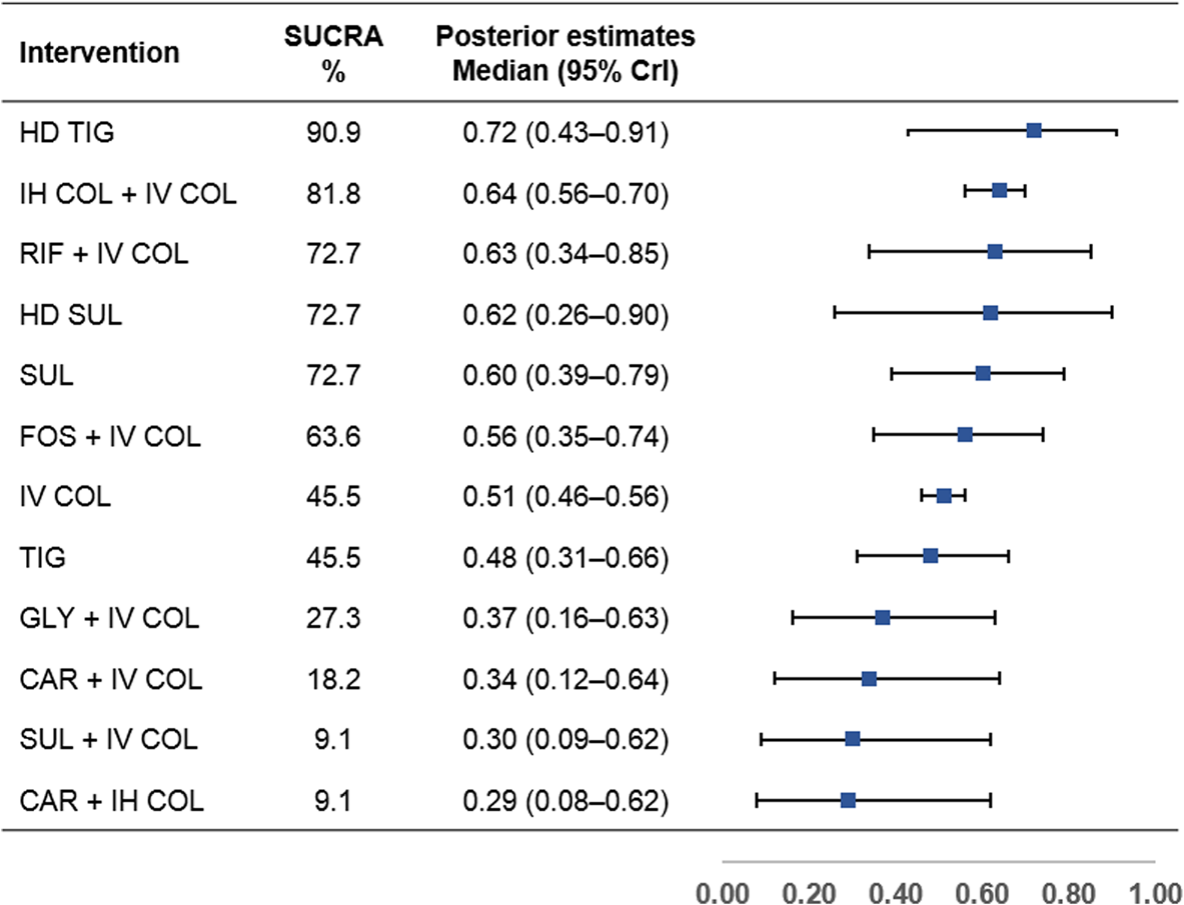
Surface under the cumulative ranking curve (SUCRA) rankings and posterior estimates of treatment effect on clinical cure. Greater SUCRA value indicates higher probability of being the best treatment for improving clinical cure. *Abbreviations*: *CAR* carbapenem (imipenem or meropenem), *COL* colistin, *CrI* credible interval, *FOS* fosfomycin, *GLY* glycopeptide (vancomycin or teicoplanin), *HD* high-dose, *IH* inhaled, *IV* intravenous, *RIF* rifampin, *SUL* sulbactam, *TIG* tigecycline

Figure 4 shows the SUCRA rank probabilities and Bayesian posterior estimates of the effect of various antimicrobial treatments on clinical cure. HD TIG ranked first for improving clinical cure (SUCRA, 90.9%; median outcome rate 0.72, 95% CrI 0.43–0.91) among the 12 antimicrobial treatments. IV COL (45.5%; 0.51, 0.46–0.56) and TIG (45.5%; 0.48, 0.31–0.66) tied for seventh place. Besides HD TIG, IH COL + IV COL (81.8%; 0.64, 0.56–0.70), RIF + IV COL (72.7%; 0.63, 0.34–0.85), HD SUL (72.7%; 0.62, 0.26–0.90), SUL (72.7%; 0.60, 0.39–0.79), and FOS + IV COL (63.6%; 0.56, 0.35–0.74) ranked higher than IV COL. SUL + IV COL (0.30, 0.09–0.62) and CAR + IH COL (0.29, 0.08–0.62) were the two lowest-ranked treatments with the same SUCRA value of 9.1%.

**Fig. 5:**
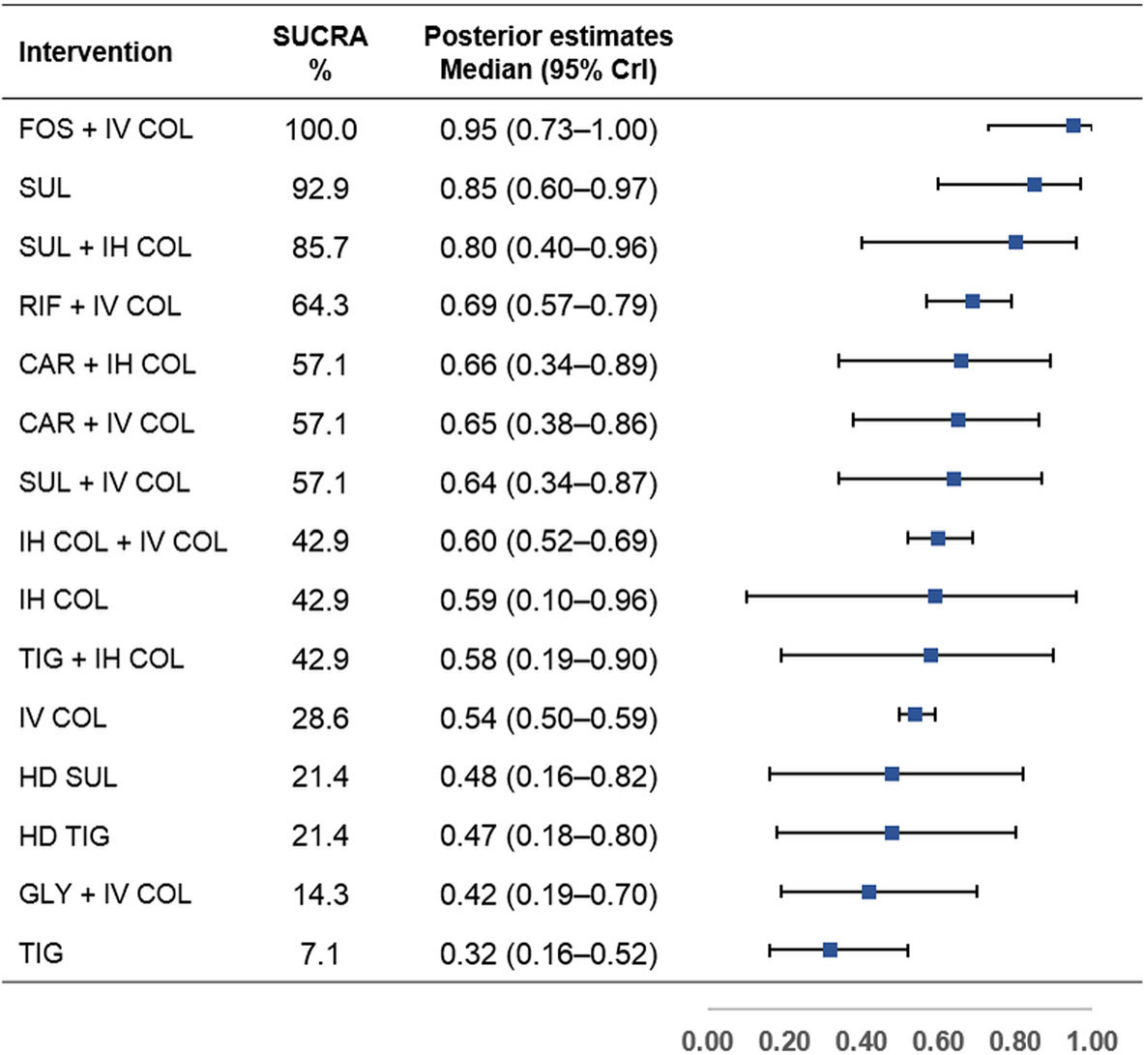
Surface under the cumulative ranking curve (SUCRA) rankings and posterior estimates of treatment effect on microbiological eradication. Greater SUCRA value indicates higher probability of being the best treatment for improving microbiological eradication. *Abbreviation*s: *CAR* carbapenem (imipenem or meropenem), *COL* colistin, *CrI* credible interval, *FOS* fosfomycin, *GLY* glycopeptide (vancomycin or teicoplanin), *HD* high-dose, *IH* inhaled, *IV* intravenous, *RIF* rifampin, *SUL* sulbactam, *TIG* tigecycline

Figure 5 presents SUCRA rank probabilities and posterior estimates of the effect of various antimicrobial treatments on microbiological eradication. FOS + IV COL ranked highest for microbiological eradication (SUCRA, 100.0%; median outcome rate 0.95, 95% CrI 0.73–1.00) among the 15 different antimicrobial treatments. SUL (92.9%; 0.85, 0.60–0.97) was next highest, followed by SUL + IH COL (85.7%; 0.80, 0.40–0.96), and RIF + IV COL (64.3%; 0.69, 0.57–0.79). CAR + IH COL (57.1%; 0.66, 0.34–0.89), CAR + IV COL (57.1%; 0.65, 0.38–0.86), and SUL + IV COL (57.1%; 0.64, 0.34–0.87) ranked fifth. IH COL + IV COL (42.9%; 0.60, 0.52–0.69), IH COL (42.9%; 0.59, 0.10–0.96), and TIG + IH COL (42.9%; 0.58, 0.19–0.90) tied for eighth place, followed by IV COL (28.6%; 0.54, 0.50–0.59). TIG (7.1%; 0.32, 0.16–0.52) was the lowest-ranked treatment among the 15 different antimicrobial treatments.

**Fig. 6:**
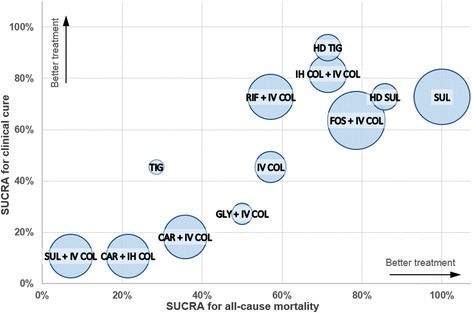
Clustered ranking plot based on surface under the cumulative ranking curve (SUCRA). The plot shows SUCRA values of twelve antimicrobial treatments, commonly included in the analyses of the following outcomes: all-cause mortality, clinical cure, and microbiological eradication. A treatment lying in the farther-right upper corner is more effective in both all-cause mortality and clinical cure than other treatments. In addition, the larger bubble size reflects the greater SUCRA value in terms of microbiological eradication. *Abbreviations*: *CAR* carbapenem (imipenem or meropenem), *COL* colistin, *FOS* fosfomycin, *GLY* glycopeptide (vancomycin or teicoplanin), *HD* high-dose, *IH* inhaled, *IV* intravenous, *RIF* rifampin, *SUL* sulbactam, *TIG* tigecycline

Figure 6 displays a clustered SUCRA ranking plot in the three dimensions of x-axis (all-cause mortality), y-axis (clinical cure), and bubble size (microbiological eradication). The plot includes 12 antimicrobial treatments, commonly included in the analyses of all three outcomes. The relative size of each bubble is in proportion to the 100% SUCRA value of FOS + IV COL. IV COL fell in the center of the plot with a relatively small bubble size. Five antimicrobial treatments (SUL, HD SUL, FOS + IV COL, IH COL + IV COL, and HD TIG) fell in farther in the right upper corner of the plot than IV COL. SUL was in the farthest-right upper position with a relatively large bubble size among the five treatments, suggesting that overall, SUL is associated with a more favorable therapeutic effect than other treatments for the three outcome variables. The bubble size of both HD SUL and HD TIG was relatively small among the top 5 treatments placed in the farthest-right upper corner, indicating that those treatments have relatively lower benefits in terms of microbiological eradication.

### Bayesian posterior probability of superiority

Table 3 presents the Bayesian probabilities of superiority to IV COL, the key comparator in NMA networks. SUL was significantly superior to IV COL for reducing all-cause mortality (OR 0.27, 95% CrI 0.06–0.91) with a 98.1% probability of superiority (*P*). TIG (OR 1.75, 95% CrI 1.09–2.81) demonstrated a higher all-cause mortality rate than IV COL with 1.0% of *P*, indicating that TIG is significantly inferior to IV COL (*P* < 2.5%) for reducing all-cause mortality. In terms of clinical cure, only IH COL + IV COL was significantly superior to IV COL (OR 1.67, 95% CrI 1.20–2.29) with a *P* of 99.9%. Except for IH COL + IV COL, there were no statistically significant differences between IV COL and the other 10 antimicrobial treatments that were evaluated in the NMA for the clinical cure outcome. FOS + IV COL (OR 15.20, 95% CrI 2.27–428.60), RIF + IV COL (1.88, 1.14–3.13), and SUL (4.82, 1.22–25.83) were significantly superior to IV COL in terms of microbiological eradication rate (Table 3; *P* = 99.8%, 99.4%, and 98.9%, respectively). On the other hand, TIG was significantly inferior to IV COL (*P* = 1.7%; OR 0.40, 95% CrI 0.16–0.94) in microbiological eradication.

**Table 3:** Bayesian NMA estimates of probability of superiority (*P*) and odds ratio (OR)

Treatment	All-cause mortality		Clinical cure		Microbiological eradication	
	*P* %	OR (95% CrI)	*P* %	OR (95% CrI)	*P* %	OR (95% CrI)
SUL	98.1*	0.27 (0.06–0.91)	80.4	1.45 (0.62–3.48)	98.9*	4.82 (1.22–25.83)
IH COL + IV COL	92.1	0.80 (0.58–1.10)	99.9*	1.67 (1.20–2.29)	90.4	1.28 (0.88–1.88)
FOS + IV COL	85.3	0.64 (0.28–1.45)	66.3	1.20 (0.52–2.72)	99.8*	15.2 (2.27–428.6)
HD SUL	74.5	0.56 (0.09–3.17)	70.1	1.54 (0.32–8.63)	38.3	0.79 (0.15–3.99)
HD TIG	65.1	0.81 (0.25–2.53)	91.8	2.48 (0.70–8.89)	36.4	0.77 (0.19–3.30)
RIF + IV COL	59.7	0.94 (0.56–1.54)	78.1	1.63 (0.48–5.54)	99.4*	1.88 (1.14–3.13)
IH COL	39.8	1.33 (0.12–13.87)	NA	NA	55.9	1.22 (0.09–19.93)
GLY + IV COL	35.3	1.13 (0.58–2.20)	14.9	0.55 (0.17–1.65)	21.6	0.62 (0.18–2.04)
TIG + IH COL	31.3	1.47 (0.26–8.32)	NA	NA	56.9	1.18 (0.18–7.40)
CAR + IV COL	21.1	1.58 (0.49–5.52)	14.2	0.50 (0.12–1.78)	78.1	1.57 (0.50–5.45)
CAR + IH COL	16.7	1.94 (0.48–8.19)	9.9	0.38 (0.08–1.64)	76.5	1.65 (0.42–7.06)
SUL + IH COL	14.2	2.32 (0.43–12.63)	NA	NA	90.4	3.37 (0.54–20.94)
SUL + IV COL	7.0	2.58 (0.71–9.88)	10.1	0.40 (0.09–1.60)	73.3	1.51 (0.42–5.75)
TIG	1.0*	1.75 (1.09–2.81)	38.7	0.89 (0.43–1.88)	1.7*	0.40 (0.16–0.94)

*Abbreviations*: *CAR* carbapenem (imipenem or meropenem), *COL* colistin, *CrI* credible interval, *FOS* fosfomycin, *GLY* glycopeptide (vancomycin or teicoplanin), *HD* high-dose, *IH* inhaled, *IV* intravenous, *NA* not available, *RIF* rifampin, *SUL* sulbactam, *TIG* tigecycline

*P > 97.5% and *P* < 2.5%, statistically significant superiority and inferiority, respectively

### Sensitivity analyses

According to the sensitivity analyses (Additional file 4: Table S5), the orders based on SUCRA values and statistical significance remained similar after removing specific studies from the analysis pool. For the first sensitivity analysis, six studies were eliminated from the original study pool because patients with other infectious diseases besides pneumonia were enrolled in those studies, though the proportion was small. Even after removing those six studies, the SUCRA rankings were comparable with those in the main analysis. In another sensitivity analysis exclusively using 14 studies in which all patients were infected by *A. baumannii*, the SUCRA value for IH COL + IV COL therapy was lower, especially for clinical cure, because most of the studies comparing IH COL + IV COL with IV COL were excluded. This sensitivity analysis suggests the robustness of our analysis by showing that concurrent infection site(s) other than the respiratory tract and the type of causative pathogen may not markedly affect the comparative effectiveness estimates of the antimicrobial treatments evaluated in this NMA.

## Discussion

To the best of our knowledge, this is the first and the most comprehensive Bayesian NMA evaluating the comparative effectiveness of various antimicrobial treatment regimens for MDR/XDR *A. baumannii* pneumonia in critically ill patients. An important finding of our study is that SUL was the most effective therapy to reduce the all-cause mortality in critically ill patients. The top five treatments with high probabilities of survival benefit were SUL (SUCRA 100.0%), HD SUL (85.7%), FOS + IV COL (78.6%), IH COL + IV COL (71.4%), and HD TIG (71.4%) (Fig. 3). Those five treatment options generally ranked high for improving clinical cure and microbiological eradication as well. However, HD TIG and HD SUL had relatively lower SUCRA values for microbiological eradication among the 15 different antimicrobial treatments. A possible explanation for those lower rankings in microbiological eradication is that, based on the published data, *A. baumannii* isolates in the two HD treatment groups were less susceptible to TIG or SUL (MIC >1 mg/L for TIG; MIC >16 mg/L for SUL, respectively) [49, 52].

The results of this study corroborate growing recent evidence that suggests SUL as a promising treatment option in the management of *Acinetobacter* infections [67–69]. Multiple clinical studies have reported that the patient group treated with SUL had a substantially low rate of mortality, ranging from 17% to 33% during approximately 2 weeks of treatment [67–70]. In addition, Oliveira et al. report that polymyxin (colistin or polymyxin B) treatment is significantly associated with higher mortality than SUL, with a relative risk of 1.52 [70]. Similarly, this Bayesian NMA demonstrated that SUL was superior to IV COL with the probabilities of superiority greater than 97.5% in terms of reducing all-cause mortality and improving microbiological eradication. However, caution needs to be exercised when interpreting and applying our findings to clinical practice, owing to the retrospective nature and relatively small sample sizes of the studies included in this NMA, and potential inherent bias, if any, in reporting the results of the original studies.

According to a recent conference report from the European Society of Intensive Care Medicine, a 4-hour infusion of SUL 3–4 g every 8 hours is recommended for severe *A. baumannii* infections involving isolates with higher MICs for SUL (≥8 mg/L) [10]. A recent pharmacodynamic modeling study conducted in healthy adults demonstrated that a 4-hour infusion of SUL 3 g every 8 hours would be an appropriate dosage regimen of SUL for less-susceptible *A. baumannii* [71]. The findings from healthy adults may not be generalizable to critically ill patients owing to pharmacokinetic alterations associated with critical illness [71, 72]. Nevertheless, it is noteworthy that prolonged-infusion dosing was found to be a much more effective strategy to achieve a high probability of target concentration attainment over a range of MICs than a dose-escalation strategy [71]. In the HD SUL treatment group in this NMA, the infusion time of HD SUL was within 1 hour even though SUL MIC for isolated *A. baumannii* was > 16 mg/L [49]. It could be inferred that a more prolonged infusion time was necessary for improving microbiological eradication, considering the time-dependent antimicrobial activity of SUL [72].

Among combination regimens evaluated in this NMA, FOS + IV COL and IH COL + IV COL had a more beneficial effect on all-cause mortality, with favorable effectiveness in clinical cure and microbiological eradication (Fig. 6). In terms of microbiological eradication, FOS + IV COL demonstrated the greatest SUCRA value in our Bayesian NMA. FOS may be an effective adjunctive therapy for pneumonia caused by MDR/XDR *A. baumannii*, considering the synergistic effect of COL and FOS in vitro [73–75]. However, owing to the paucity of clinical data evaluating the efficacy and safety of FOS + IV COL, adjunctive FOS should be used with caution until clinical studies adequately confirm its promising effect in patients with severe pneumonia. According to the guideline recently updated by the Infectious Diseases Society of America, adjunct IH COL therapy is suggested for the treatment of HAP/VAP due to *A. baumannii* that is sensitive only to COL, particularly for patients with insufficient response to IV COL monotherapy [76]. Similarly, in this NMA, the probability of superiority analysis showed that IH COL + IV COL yielded additional therapeutic superiority in terms of clinical cure, and comparable effectiveness in other outcomes, compared with IV COL.

Besides IH COL, several aerosolized antimicrobial agents including amikacin, tobramycin, and fosfomycin were evaluated for the treatment of gram-negative pneumonia in previous studies comparing the efficacy of IH antimicrobial adjunct therapy to various IV antimicrobial agents with that of IV monotherapy or IH placebo [77–80]. However, antimicrobial agents evaluated in those studies could not be included as treatment nodes in our NMA to construct a single connected network. Furthermore, non-MDR/XDR *A. baumannii* pneumonia patients were largely included in those studies. Of note, more evidence is required to determine the appropriate administration method of IH antimicrobials for optimal clinical benefit in patients with pneumonia because the delivery devices, dosing, ventilator settings, and endotracheal tube size may affect the therapeutic response [76, 81].

There are a few limitations in this meta-analysis. The major limitation of this NMA is the absence of aerosolized antimicrobials other than IH COL as aforementioned. Another important limitation is that the analysis was mostly based on retrospective studies (16 out of 23 studies in total). Considering the retrospective nature of most included studies, this NMA should be viewed as a hypothesis-generating study rather than definitive clinical evidence, despite rigorous quality assessments. The safety profiles of all antimicrobial treatments were not evaluated due to insufficient data on adverse drug reactions and substantial differences between studies in the baseline laboratory values or organ function parameters. In addition, the novel antimicrobial treatments with potential activity against A. *baumannii*, such as vabomere, plazomicin, cefiderocol and eravacycline, were not included in this NMA owing to absence of published data on the patients with MDR/XDR *A. baumannii* pneumonia [82–84]. It remains to be determined whether any of these agents may have a role in this clinically important infection. Additionally, this NMA could not clearly address the role of combination therapy over single-agent therapy. In fact, the purpose of this NMA was not to specifically compare the effectiveness between combination therapy and monotherapy but to evaluate the overall comparative effectiveness of different antimicrobial treatment options. Last, variability in identifying the causative pathogen among the included studies could not completely distinguish colonization from infection in HAP and VAP. Clinical studies more robustly detecting the true infectious organism in HAP or VAP may be necessary to accurately and precisely determine the effects of antimicrobial treatments for MDR/XDR *A. baumannii* pneumonia.

## Conclusions

This Bayesian NMA provides clinically meaningful evidence to aid clinicians in selecting the optimal antimicrobial regimen for the treatment of critically ill patients with MDR/XDR *A. baumannii* pneumonia. SUL appears to be the best treatment among fifteen different antimicrobial regimens investigated for survival benefit. Apart from SUL, FOS + IV COL, HD SUL, IH COL + IV COL, and HD TIG therapy may be appropriate alternative treatment options. The comparative results of our analysis should be tailored to the antimicrobial susceptibility testing result of each individual patient when making treatment decisions. Appropriate and well-controlled studies would be necessary to prospectively verify the findings of our current study in patients with nosocomial pneumonia.

## Additional files

Additional file 1: Table S1. PRISMA checklist of items to include when reporting a systematic review and a network meta-analysis. (DOCX 20 kb)

Additional file 2: Table S2. Risk of bias assessment of randomized controlled trials. **Table S3.** Risk of bias assessment of observational studies. (DOCX 20 kb)

Additional file 3: Table S4. Inconsistency assessment. (DOCX 14 kb)

Additional file 4: Table S5. Results of sensitivity analyses. (DOCX 17 kb)

## Abbreviations

*AB*: *Acinetobacter baumannii*
APACHE II: Acute Physiology and Chronic Health Evaluation II
CAR: Carbapenem
CI: Confidence interval
COL: Colistin
CR: Carbapenem-resistant
CrI: Credible interval
DIC: Deviance information criterion
FOS: Fosfomycin
GLY: Glycopeptide
HAP: Hospital-acquired pneumonia
HD: High dose
IH: Inhaled
IMI: Imipenem
IV: Intravenous
*KP*: *Klebsiella pneumoniae*
MCMC: Markov chain Monte Carlo
MDR: Multidrug-resistant
MERO: Meropenem
MIU: Million international units
n/N: Number of outcome events/number of patients in each intervention
NR: Not reported
OR: Odds ratio
*PA*: *Pseudomonas aeruginosa*
PRISMA: Preferred Reporting Items for Systematic Reviews and Meta-analyses
RIF: Rifampin
SAPS: Simplified acute physiology score
SOFA: Sepsis-related organ failure assessment score
SUCRA: Surface under the cumulative ranking curve
SUL: Sulbactam
TDM: Therapeutic drug monitoring
TEI: Teicoplanin
TIG: Tigecycline
VAN: Vancomycin
VAP: Ventilator-associated pneumonia
XDR: Extensively drug-resistant
