# Supplementary material for: Antimicrobials for the treatment of drug-resistant Acinetobacter baumannii pneumonia in critically ill patients: a systemic review and Bayesian network meta-analysis
Source: Crit Care. 2017 Dec 20;21:319. doi: 10.1186/s13054-017-1916-6 (PMC5738897; doi:10.1186/s13054-017-1916-6)
Supplement: Supplementary file 2 — Risk of bias assessment of randomized controlled trials. Table S3. Risk of bias assessment of observational studies. (DOCX 20 kb) [file 13054_2017_1916_MOESM2_ESM.docx]

**Table S2. Risk of bias assessment of randomized controlled trials.**

| **First author of each study** | **Adequate sequence generation?** | **Allocation concealment?** | **Blinding of participants and personnel?** | **Blinding of outcome assessment?** | **Incomplete outcome data addressed?** | **Free of selective reporting?** | **Free of other bias (no funding)?** |
| --- | --- | --- | --- | --- | --- | --- | --- |
| Abdellatif | Y | U | N | U | Y | Y | Y |
| Aydemir | Y | U | Y | U | Y | Y | Y |
| Durante-Mangoni | Y | Y | Y | U | Y | Y | Y |
| Sirijatuphat | Y | U | N | U | Y | Y | Y |

Abbreviations: *Y* Yes (i.e. low risk of bias), *N* No (i.e. high risk of bias), *U* Unclear (i.e. insufficient information to permit judgement of ‘low risk’ or ‘high risk’)

**Table S3. Risk of bias assessment of observational studies.**

| **First author of each study** | **Selection bias** | | | | **Comparability bias**  **on study design or analysis** | **Outcome bias** | | | **Total score** |
| --- | --- | --- | --- | --- | --- | --- | --- | --- | --- |
|  | **Exposed**  **cohort** | **Non-exposed cohort** | **Ascertainment of exposure** | **Outcome of interest** |  | **Assessment of outcome** | **Length of follow-up** | **Adequacy of follow-up** |  |
| Amin | ***** | ***** | ***** | ***** |  | ***** | ***** | ***** | 7 |
| Betrosian | ***** | ***** | ***** | ***** | ***** | ***** | ***** | ***** | 8 |
| Chuang | ***** | ***** | ***** | ***** | ***** | ***** | ***** | ***** | 8 |
| De Pascale | ***** | ***** | ***** | ***** |  | ***** | ***** | ***** | 7 |
| Demirdal | ***** | ***** | ***** | ***** |  | ***** | ***** | ***** | 7 |
| Doshi | ***** | ***** | ***** | ***** |  | ***** | ***** | ***** | 7 |
| Frantzeskaki | ***** | ***** | ***** | ***** |  | ***** | ***** | ***** | 7 |
| Garnacho Montero | ***** | ***** | ***** | ***** |  | ***** | ***** | ***** | 7 |
| Hsieh | ***** | ***** | ***** | ***** | ***** | ***** | ***** | ***** | 8 |
| Kalin | ***** | ***** | ***** | ***** |  | ***** | ***** | ***** | 7 |
| Khawcharoenporn | ***** | ***** | ***** | ***** | ***** | ***** | ***** | ***** | 8 |
| Kim | ***** | ***** | ***** | ***** |  | ***** | ***** | ***** | 7 |
| Kofteridis | ***** | ***** | ***** | ***** |  | ***** | ***** | ***** | 7 |
| Korbila, | ***** | ***** | ***** | ***** |  | ***** | ***** | ***** | 7 |
| Kwon | ***** | ***** | ***** | ***** |  | ***** | ***** | ***** | 7 |
| Petrosillo | ***** | ***** | ***** | ***** | ***** | ***** | ***** | ***** | 8 |
| Tumbarello | ***** | ***** | ***** | ***** | ****** | ***** | ***** | ***** | 9 |
| Yilmaz | ***** | ***** | ***** | ***** |  | ***** | ***** | ***** | 7 |
| Zalts | ***** | ***** | ***** | ***** | ***** | ***** | ***** | ***** | 8 |

* = one score, **= two score
