# Supplementary material for: Antimicrobials for the treatment of drug-resistant Acinetobacter baumannii pneumonia in critically ill patients: a systemic review and Bayesian network meta-analysis
Source: Crit Care. 2017 Dec 20;21:319. doi: 10.1186/s13054-017-1916-6 (PMC5738897; doi:10.1186/s13054-017-1916-6)
Supplement: Supplementary file 3 — Inconsistency assessment. (DOCX 14 kb) [file 13054_2017_1916_MOESM3_ESM.docx]

**Table S4. Inconsistency assessment.**

|  |  | | All-cause mortality | Clinical Cure | Microbiological eradication |
| --- | --- | --- | --- | --- | --- |
| Entire network (design by treatment) Inconsistency | | χ2 | 1.76 | 0.37 | 0.19 |
|  |  | p-value* | 0.18 | 0.54 | 0.66 |

*If the p-value is less than 0.05, the null hypothesis (no inconsistency) is rejected.
