# Supplementary material for: Antimicrobials for the treatment of drug-resistant Acinetobacter baumannii pneumonia in critically ill patients: a systemic review and Bayesian network meta-analysis
Source: Crit Care. 2017 Dec 20;21:319. doi: 10.1186/s13054-017-1916-6 (PMC5738897; doi:10.1186/s13054-017-1916-6)
Supplement: Supplementary file 4 — Results of sensitivity analyses. (DOCX 17 kb) [file 13054_2017_1916_MOESM4_ESM.docx]

**Table S5. Results of sensitivity analyses.**

| **All-cause mortality** | **Main analysis** | | | **Sensitivity analysis 1: Pneumonia 100%** | | | **Sensitivity analysis 2: *A. baumannii* 100%** | | |
| --- | --- | --- | --- | --- | --- | --- | --- | --- | --- |
|  | SUCRA (%) | Rank | Probability of superiority (%) | SUCRA (%) | Rank | Probability of superiority (%) | SUCRA  (%) | Rank | Probability of superiority (%) |
| SUL  HD SUL  FOS + IV COL  IH COL + IV COL  HD TIG  RIF + IV COL  IV COL  GLY + IV COL  IH COL  CAR + IV COL  TIG + IH COL  TIG  CAR + IH COL  SUL + IH COL  SUL + IV COL | 100.0  85.7  78.6  71.4  71.4  57.1  57.1  50.0  42.9  35.7  35.7  28.6  21.4  14.3  7.1 | 1 (best)  2  3  4  4  6  6  8  9  9  11  12  13  14  15 (worst) | 98.1  74.5  85.3  92.1  65.1  59.7  Comparator  35.3  39.8  21.1  31.3  1.0  16.7  14.2  7.0 | 100.0  83.3  -  75.0  66.7  83.3  58.3  -  50.0  33.3  41.7  25.0  25.0  16.7  8.3 | 1 (best)  2  -  4  5  2  6  -  7  9  8  10  10  12  13 (worst) | 98.3  73.4  -  91.9  76.2  78.7  Comparator  -  41.3  22.4  33.6  29.6  18.6  16.7  7.2 | 100.0  83.3  -  66.7  75.0  75.0  58.3  -  41.7  33.3  41.7  41.7  25.0  16.7  8.3 | 1 (best)  2  5  3  3  6  -  7  10  7  7  11  12  13 (worst) | 98.1  73.9  -  92.1  75.3  78.4  Comparator  -  39.8  22.1  32.3  28.9  17.3  15.6  7.9 |
| **Clinical Cure** | **Main analysis** | | | **Sensitivity analysis 1: Pneumonia 100%** | | | **Sensitivity analysis 2: *A. baumannii* 100%** | | |
|  | SUCRA (%) | Rank | Probability of superiority (%) | SUCRA (%) | Rank | Probability of superiority (%) | SUCRA (%) | Rank | Probability of superiority (%) |
| HD TIG  IH COL + IV COL  RIF + IV COL  HD SUL  SUL  FOS + IV COL  IV COL  TIG  GLY + IV COL  CAR + IV COL  SUL + IV COL  CAR + IH COL | 90.9  81.8  72.7  72.7  72.7  63.6  45.5  45.5  27.3  18.2  9.1  9.1 | 1 (best)  2  3  3  3  6  7  8  9  10  11(worst)  11 (worst) | 91.8  99.9  78.1  70.1  80.4  66.3  Comparator  38.7  14.9  14.2  10.1  9.9 | 88.9  66.7  77.8  77.8  66.7  -  44.4  44.4  -  22.2  11.1  11.1 | 1 (best)  4  2  2  4  -  6  6  -  8  9 (worst)  9 (worst) | 91.3  99.8  78.1  70.5  80.9  -  Comparator  46.9  -  15.3  7.1  10.5 | -  33.3  88.9  77.8  77.8  77.8  55.6  44.4  22.2  11.1  11.1  - | -  7  1 (best)  2  2  2  5  6  8  9  9 (worst)  - | -  18.5  77.3  70.5  80.8  71.8  Comparator  39.3  14.9  13.6  9.4  - |
| **Microbiological eradication** | **Main analysis** | | | **Sensitivity analysis 1: Pneumonia 100%** | | | **Sensitivity analysis 2: *A. baumannii* 100%** | | |
|  | SUCRA (%) | Rank | Probability of superiority (%) | SUCRA  (%) | Rank | Probability of superiority (%) | SUCRA  (%) | Rank | Probability of superiority (%) |
| FOS + IV COL  SUL  SUL + IH COL  RIF + IV COL  CAR + IH COL  CAR + IV COL  SUL + IV COL  IH COL + IV COL  IH COL  TIG + IH COL  IV COL  HD SUL  HD TIG  GLY + IV COL  TIG | 100.0  92.9  85.7  64.3  57.1  57.1  57.1  42.9  42.9  42.9  28.6  21.4  21.4  14.3  7.1 | 1 (best)  2  3  4  5  5  5  8  8  8  11  12  12  14  15 (worst) | 99.8  98.9  90.4  99.4  76.5  78.1  73.3  90.4  55.9  56.9  Comparator  38.3  36.4  21.6  1.7 | -  100.0  83.3  66.7  58.3  58.3  50.0  50.0  33.3  33.3  33.3  16.7  50.0  -  16.7 | -  1 (best)  2  3  4  4  6  6  8  8  8  11  6  -  11 (worst) | -  98.8  88.1  81.0  72.2  74.7  71.0  90.5  52.1  52.7  Comparator  38.2  64.6  -  26.3 | 100.0  88.9  -  66.7  -  55.6  55.6  33.3  -  -  33.3  22.2  -  11.1  0.0 | 1 (best)  2  -  3  -  4  4  6  -  -  6  8  -  9  10 (worst) | 99.8  98.8  -  99.3  -  75.5  72.3  46.5  -  -  Comparator  37.9  -  21.5  1.8 |

Abbreviations: *A. baumannii Acinetobacter baumannii*, *CAR* carbapenem (imipenem or meropenem), *COL* colistin, *FOS* fosfomycin, *GLY* glycopeptide (vancomycin or teicoplanin), *HD* high dose, *IH* inhaled, *IV* intravenous, *RIF* rifampin, *SUCRA* surface under the cumulative ranking curve, *SUL* sulbactam, *TIG* tigecycline.
